# Simple X-ray Metric Predicts Postoperative Course After Spontaneous Pneumothorax Surgery

**DOI:** 10.7759/cureus.104426

**Published:** 2026-02-28

**Authors:** Eyüp Halit Yardimci, Nur Simge Kokles

**Affiliations:** 1 Thoracic Surgery, Marmara University, Istanbul, TUR; 2 Thoracic Surgery, Dr. Siyami Ersek Research and Training Hospital, Istanbul, TUR

**Keywords:** body type, chest x-ray (cx-ray), pneumothorax (ptx), prolonged air leak, radiologic findings

## Abstract

Background: Predicting postoperative outcomes in patients with spontaneous pneumothorax (SP) remains clinically valuable. This study investigates whether a radiological thoracic (RT) index, calculated as the chest height-to-width ratio on posteroanterior (PA) chest radiographs (CXR), can serve as a predictor after SP surgery.

Methods: In this retrospective study, 195 patients who underwent surgery for SP were included. Patients were grouped according to surgery reasons as recurrence (n=101), preoperative persistent air leak (PAL) (n=58), and bilateral pneumothorax history (n=36). The RT index was calculated on PA CXRs using the involved side (right side for bilateral cases). Comparisons were made between patient groups, and linear regression was used to determine independent predictors of the RT index.

Results: The mean RT index was 2.41±0.33. The RT index was significantly higher in patients with chest tube duration ≥5 days (2.49±0.34 vs. 2.34±0.31, p<0.001). Age was negatively correlated with the RT index (r=-0.459, p<0.001), while chest tube duration after surgery positively correlated (r=0.170, p=0.017). Multivariate regression identified age and postoperative PAL as independent predictors of the RT index (p<0.001). No significant difference in the RT index was found among recurrence, preoperative PAL, or bilateral pneumothorax history groups.

Conclusion: The RT index derived from routine CXRs may help predict prolonged chest tube duration in patients undergoing surgery for SP. This simple radiological metric could contribute to perioperative risk stratification.

## Introduction

Spontaneous pneumothorax (SP) remains a frequent cause of emergency thoracic admissions, particularly affecting adolescent and young adult males without underlying pulmonary disease. While the etiology of SP is often attributed to subpleural blebs or bullae, the pathophysiological contributors to recurrence or prolonged air leak following surgical treatment are multifactorial and not fully understood.

Recent literature has underscored the role of thoracic morphology in predisposing individuals to SP. The so-called "pneumothorax body type," characterized by tall stature, low body mass index (BMI), and increased chest verticality, has been associated with increased susceptibility to initial and recurrent pneumothorax episodes [1]. Moreover, anatomical thoracic features observed on computed tomography (CT) or chest radiography (CXR) may have predictive value for disease progression and postoperative outcomes. For example, Akamine et al. reported that interpleural distance measured on CXR correlates with the risk of persistent air leak (PAL) in primary SP patients [2]. Similarly, Asano et al. identified specific radiographic signs such as diaphragmatic tenting and pleural cavities as potential markers for recurrence after thoracoscopic surgery [3]. Mitani et al. further noted that increased height velocity and lower BMI in adolescents may correlate with asymptomatic primary SP occurrence, emphasizing anatomical predisposition [1].

Despite these insights, predictive tools based on standard CXRs are rarely integrated into clinical practice, particularly those relying on accessible, quantitative anatomical ratios. Posteroanterior (PA) CXRs are almost universally obtained at initial presentation and preoperatively, offering an opportunity for structural thoracic assessment without additional cost or radiation exposure. While artificial intelligence (AI) models have recently demonstrated improved detection accuracy for pneumothorax using thoracic shape and position data [4], little is known about whether manually derived chest dimensions can help anticipate postoperative outcomes.

In this study, we investigate the utility of a simple radiographic index, calculated as the ratio of vertical thoracic height to horizontal thoracic width (RT index), measured manually on preoperative PA CXRs. We aimed to evaluate whether the radiological thoracic (RT) index, calculated as the chest height-to-width ratio on preoperative PA CXRs, predicts postoperative outcomes, particularly prolonged chest tube duration, in patients undergoing surgery for SP. We hypothesized that thoracic geometry, as quantified by the RT index, may serve as a useful adjunct in surgical decision-making and patient counseling in cases of SP.

## Materials and methods

Study design and patients

The study is designed as a retrospective cohort. A total of 342 patients who underwent surgery for SP at a single center between January 2014 and December 2023 were evaluated using hospital records. Inclusion criteria were male gender, age ≥18 years, and availability of preoperative PA CXRs. Female gender, non-SP cases (e.g., trauma), pediatric cases, emphysema surgeries like lung volume reductions, and cases without proper hospital records were excluded. The study population consisted of 195 patients. All patients were operated on via minimally invasive methods. Lung tissue-preserving wedge resection and partial parietal pleurectomy to prevent recurrence was the standard surgical procedure. Patients had a single chest tube after surgery. The chest tube was removed following 24 hours of cessation of air leak and a radiologically expanded lung on CXR. Air leak was evaluated by observational methods; digital devices were unavailable and not used. Based on preoperative clinical presentation, patients were categorized into three subgroups: those with a history of recurrence, those who experienced preoperative PAL (defined as air leak longer than five days), and those with a prior history of bilateral pneumothorax.

Radiological assessment

**Figure 1:**
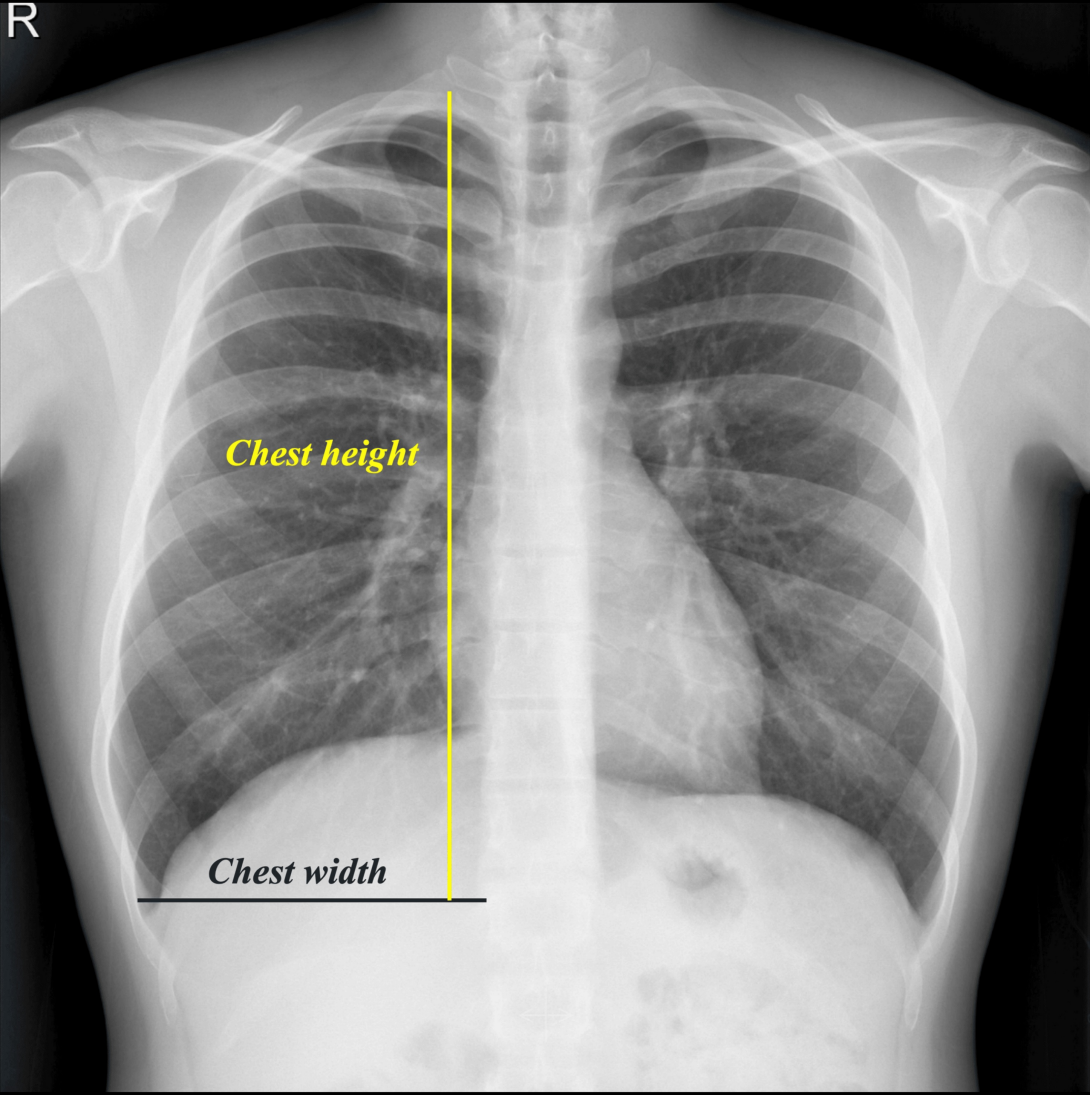
Measuring method of chest height and width from a PA CXR PA, posteroanterior; CXR, chest radiography

For each patient, a radiological thoracic (RT) index was calculated as the ratio of vertical chest height to horizontal chest width on a PA CXR, as shown in Figure 1.

Chest height was defined as the distance from the lung apex to the costophrenic angle, and chest width as the distance between the medial borders of the ribs from the level of the costophrenic angle to the mediastinal opacity border. For patients with a history of bilateral pneumothorax, the right side was used for measurement; otherwise, the affected side was used.

Clinical data and outcomes

Demographic data, operative side, pathology results (presence of blebs/bullae) (pathology results were not available for 20 cases), preoperative CT findings (presence of blebs/bullae), and postoperative outcomes, including chest tube duration to demonstrate an objective cessation time of a probable air leak postoperatively, were recorded. The relevance of the RT index data was evaluated statistically for these criteria.

Statistical analysis

Statistical analysis was performed using IBM SPSS version 25.0 (IBM Corp., Armonk, NY, USA). The distribution of variables was assessed with the Kolmogorov-Smirnov test, and homogeneity of variance was evaluated with Levene’s test. Group comparisons were performed using independent samples t-tests and one-way ANOVA, with Bonferroni correction for post-hoc comparisons. Correlations were analyzed using Spearman’s correlation coefficient. Multivariate linear regression (stepwise) was used to identify independent predictors of the RT index. A p-value <0.05 was considered statistically significant. Pathological bleb/bulla/emphysema data were incomplete for a subset of patients; therefore, analyses involving this variable, including regression models, were conducted using complete-case methodology without data imputation.

## Results

The study included 195 patients with a mean age of 34.6 years (±14.6), ranging from 18 to 89. Of these, 100 patients underwent right-sided and 95 left-sided operations. The recurrence group had 101 cases (51.8%), the PAL group had 58 cases (29.7%), and the history of bilateral pneumothorax group had 36 cases (18.5%). Demographics and group distributions are provided in Table 1.

**Table 1:** Demographic and clinical characteristics of the patients *Data are presented as mean ± standard deviation. **Data are presented as median (minimum-maximum). ^a^Pathology reports were only available for 175 cases. PAL, persistent air leak; CT, computed tomography; RT, radiological thoracic

Characteristics	n=195
Age (years)*	34.6±14.6
Range (years)	18-89
Operation side	
Right	100 (51.3%)
Left	95 (48.7%)
Indication for surgery	
Recurrence	101 (51.8%)
PAL	58 (29.7%)
Bilateral history	36 (18.5%)
Pathology (bleb/bulla/emphysematous changes)^a^	
Absent	21 (12.0%)
Present	154 (88.0%)
Bleb/bulla on CT	
Absent	12 (6.2%)
Present	183 (93.8%)
Postoperative chest tube duration (days)**	5 (2-30)
Chest height (mm)*	300.84±27.25
Chest width (mm)*	125.88±11.63
RT index (height/width)*	2.41±0.33

The mean RT index across the cohort was 2.41 (±0.33), with a range from 1.64 to 3.39. The median chest tube duration was five days, with a minimum of two and a maximum of 30 days.

No statistically significant difference in the RT index was found among the clinical subgroups of recurrence, preoperative PAL, or bilateral pneumothorax history (p=0.718). Similarly, the RT index did not differ significantly by pathological findings (p=0.828) or the presence of blebs or bullae on CT imaging (p=0.555) (Table 2).

**Table 2:** RT index according to demographic and clinical characteristics of the patients Data are presented as mean ± standard deviation. *Pathology reports were only available for 175 cases. ** Among patients with negative CT findings (bleb/bulla), "Pathology positive" denotes the presence of bleb or bulla on histopathological examination, whereas "Pathology negative" indicates the absence of these findings. ^a^One-way ANOVA. ^b^Student’s t-test. PAL, persistent air leak; CT, computed tomography; RT, radiological thoracic

Characteristics	n	RT index (mean ± standard deviation)	Test statistic	p-value
Indication for surgery			F=0.332	0.718^a^
Recurrence	101	2.42±0.34		
PAL	58	2.39±0.37		
Bilateral history	36	2.44±0.27		
Pathological bleb/bulla/emphysematous changes*			t=-0.508	0.612^b^
Absent	21	2.40±0.36		
Present	154	2.44±0.33		
CT finding negative (bleb/bulla)**			t=0.440	0.669^b^
Pathology negative	4	2.60±0.15		
Pathology positive	8	2.51±0.35		
Postoperative chest tube duration			t=-3.230	<0.001^b^
≤4 days	96	2.34±0.31		
≥5 days	99	2.49±0.34		

**Figure 2:**
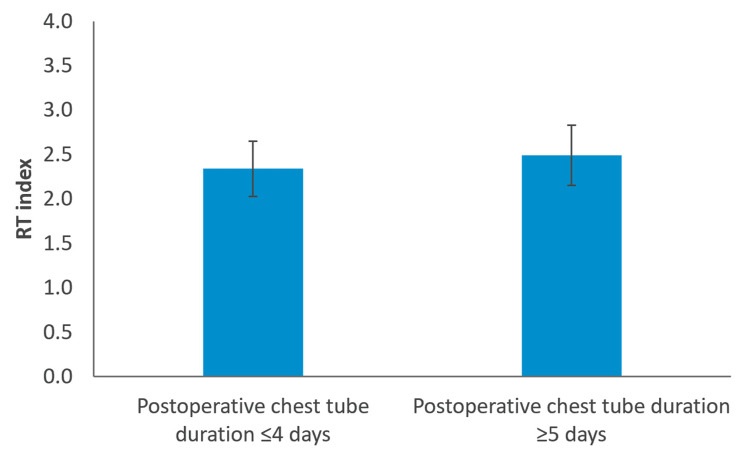
RT index comparison among postoperative chest tube duration groups Comparison of mean RT index values between patients with postoperative chest tube duration ≤4 days (n=96) and those with postoperative chest tube duration ≥5 days (n=99). Bars represent mean RT index values, and error bars indicate ±1 standard deviation. Differences between groups were analyzed using the independent samples Student’s t-test (p<0.001). RT, radiological thoracic

When patients were grouped by air leak duration, those with a chest tube stay of five days or more had significantly higher RT index values compared to those with air leak durations of four days or less (2.49±0.34 vs. 2.34±0.31; p<0.001) (Figure 2, Table 2).

**Figure 3:**
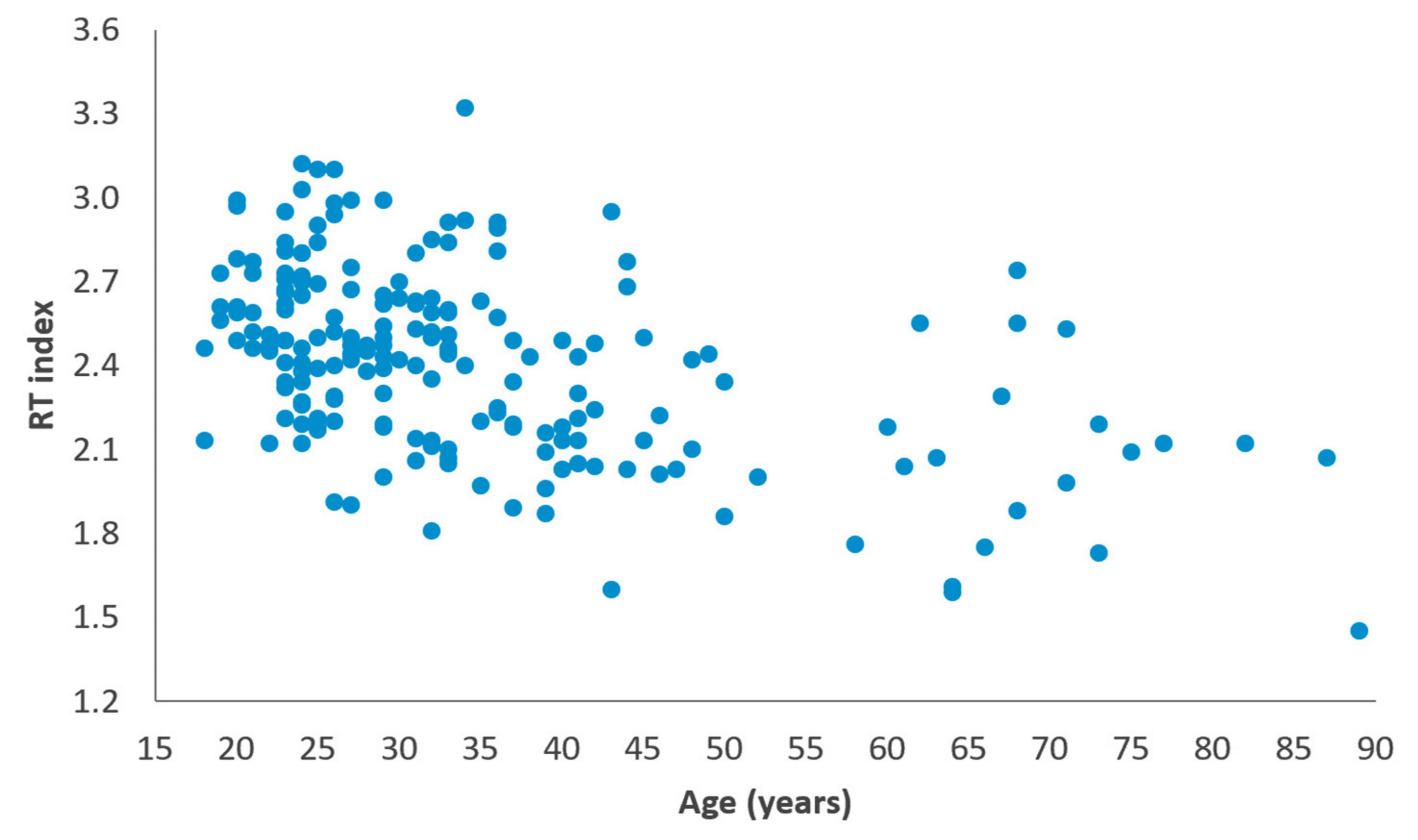
RT index distribution according to age Scatter plot showing the association between age (years) and RT index (n=195). The correlation was assessed using Spearman’s rank correlation (ρ=-0.459, p<0.001). RT, radiological thoracic

Correlation analysis revealed a moderate negative relationship between the RT index and age (r=-0.459, p<0.001), indicating that RT index values tend to decrease with increasing age. The RT index distribution according to age is shown in Figure 3. A statistically significant positive correlation was observed between the RT index and chest tube duration (r=0.170, p=0.017). In the multivariate linear regression model, both younger age (B=-0.012, p<0.001) and prolonged air leak after surgery (B range: 0.117-0.139, p<0.001) remained independent predictors of a higher RT index (Table 3). Other variables, such as having blebs/bullae in pathology results or preoperative CT findings and the side of surgery, were not significantly associated with the index.

**Table 3:** Results of the multivariate linear regression model evaluating the RT index PAL, persistent air leak; RT, radiological thoracic

	Regression coefficient	95% CI		t-statistic	p-value
Age	-0.012	-0.015	-0.009	-7.835	<0.001
Left side	0.063	-0.023	0.150	1.446	0.150
Recurrence	0.041	-0.071	0.153	0.716	0.475
PAL	0.133	0.004	0.263	2.031	0.044
Chest tube stay ≥5 days	0.117	0.032	0.203	2.711	0.007
Bleb/bullae/emphysema presence at pathology	0.050	-0.080	0.181	0.765	0.445

## Discussion

This study demonstrates that a simple radiological metric, the RT index, derived from routine PA CXRs, is a significant predictor of prolonged chest tube duration following surgery for SP. We found that patients with a vertically elongated thoracic morphology (higher RT index) were significantly younger and more likely to experience PAL. These findings reinforce the clinical utility of analyzing thoracic geometry to stratify perioperative risk. While advanced imaging and computational methods are evolving, the prognostic value of basic thoracic geometry remains fundamental. Our results align with emerging insights from AI research. Lee et al. recently reported that thoracic geometry and projection angles significantly influence the accuracy of AI-based pneumothorax detection, suggesting that intrinsic anatomical shape holds diagnostic and potentially prognostic patterns beyond simple air accumulation [4]. However, implementing such complex AI models requires significant infrastructure. In contrast, our study demonstrates that a manually derived metric, the RT index, can offer similar prognostic utility by quantifying this "pneumothorax-prone" geometry. This concept is further supported by Akamine et al., who showed that a simple measurement of "interpleural distance" on a standard chest X-ray was an independent predictor of PAL in first-episode patients, outperforming more complex indices [2].

The association we observed between a high RT index and younger age aligns with the well-described "pneumothorax body type," characterized by a tall, thin stature and a flat thorax [5]. Nonomura et al. recently highlighted that teenage patients with SP exhibit distinct body shape characteristics compared to those in their 20s, specifically noting a more vertically elongated thorax that may not keep pace with visceral pleural development [5]. Similarly, Mitani et al. identified that rapid height growth and low BMI are significant risk factors for SP, suggesting that skeletal elongation creates a mechanical environment prone to pleural failure [1]. Our finding that the RT index correlates negatively with age (r=-0.459) supports the hypothesis that this "vertical" thoracic morphology is most pronounced during the growth phase and serves as a predisposing anatomic factor for more complex postoperative courses.

The most clinically relevant finding of our study is the independent association between a high RT index and prolonged postoperative chest tube duration (≥5 days). The mechanism linking thoracic elongation to prolonged air leak likely involves a mechanical mismatch between the lung and the chest wall. Chopra et al. described "lung-thoracic cavity shape/size mismatch" as a key driver of pressure-dependent pneumothorax and air leaks, where the lung parenchyma struggles to conform to the chest wall, preventing pleural apposition [6]. In a vertically elongated thorax (high RT index), the apical pleural stress is significantly increased [5], and the remaining lung after resection may have difficulty expanding to fill the apex, thereby perpetuating the air leak. This concept is supported by Bertolaccini et al., who emphasized that variations in chest wall shape directly influence pleural stress distribution and fluid dynamics, potentially hindering the sealing of alveolar-pleural fistulas [7].

Modern pneumothorax surgery usually results in shorter chest tube stays, but the optimal timing of postoperative chest tube removal is still not standardized. Our study's chest tube removal period sometimes exceeded the expected periods. The reasons for this seem to be the presence of apical residual spaces and surgeons' preference to wait for adequate pleurodesis after partial pleurectomy. A recent meta-analysis suggests that early chest tube removal (before 2.5 days) may be associated with a slightly higher recurrence rate after surgery [8]. Gaunt et al.'s study also showed that the presence of postoperative residual apical space is related to longer chest tube stays and higher recurrence rates [9]. According to Boyle's law, there is an inverse relationship between pressure and volume. The apical pleural space is anatomically conical, and this space is difficult to obliterate [10]. This seems to be the reason for the need for longer chest tube stays after pneumothorax surgery without technical complications.

Furthermore, our results mirror findings in the literature regarding BMI and air leaks, as the RT index can be considered a radiological surrogate for the "thin" phenotype. Pompili et al. and Orsini et al. identified low BMI as an independent risk factor for prolonged air leak in their risk scoring systems for lobectomy patients [11]. While their studies focused on lung resection for malignancy, the physiological principle likely remains consistent for SP: patients with a leaner, more elongated thoracic frame (high RT index) lack the extrapleural fat or compliant soft tissue that might otherwise assist in sealing air leaks or reducing pleural space volume. Additionally, Cattoni et al. identified postoperative prolonged air leak as the only independent risk factor for recurrence in a large multicentric series [12]. Therefore, identifying patients with a high RT index preoperatively could alert surgeons to a higher risk of PAL and, consequently, a potentially higher risk of long-term recurrence.

The utility of using a simple CXR-based metric is also supported by recent literature. While CT scans provide detailed anatomical data, they expose young patients to radiation and are not always cost-effective for initial screening. Akamine et al. demonstrated that a simple measurement of "interpleural distance" on a chest X-ray was an independent predictor of PAL in patients with a first episode of SP [2]. Our study extends this concept by showing that the intrinsic shape of the thorax (RT index), rather than just the size of the pneumothorax, holds prognostic value. This allows for risk stratification using the most basic and universally available diagnostic tool.

Limitations

This study is retrospective; therefore, prospective validation is needed. Although radiology supervision was provided and all CXR measurements were performed by the same individual, manual measurement may still be subject to interobserver variability, despite reflecting real-world clinical practice. The study population was limited to operated cases, and results may not generalize to patients managed conservatively. Additionally, incomplete pathological bleb, bulla, or emphysema data may have introduced potential bias in analyses involving this variable. Lastly, although we hypothesized anatomical mechanisms, we did not assess pulmonary compliance or perform lung function tests.

## Conclusions

This study identifies an easily calculable CXR metric as a significant predictor of postoperative chest tube duration in patients undergoing surgery for SP. A vertically elongated thorax predicts prolonged chest tube duration, likely due to lung-chest wall mismatch and increased apical stress. This simple tool can aid surgeons in preoperative counseling and managing expectations regarding hospital length of stay, particularly in younger patients with the classic pneumothorax body habitus.
